# A phase 1 trial of HPV16 E7 T-cell receptor-engineered T cells in patients with relapsed/refractory HPV16-positive cancers (KITE-439 trial)

**DOI:** 10.3389/fonc.2026.1809354

**Published:** 2026-05-21

**Authors:** Kedar Kirtane, Jiaxin Niu, George Blumenschein, Erminia Massarelli, Glenn J. Hanna, Sylvia Lee, Michael R. Bishop, Gottfried E. Konecny, Daqin Mao, Yan Zheng, Katherine Rodriguez, Jenny J. Kim, Chad Williams, Colleen Schweitzer, Sabina Adhikary, A. Scott Jung, Christopher A. Klebanoff

**Affiliations:** 1 Department of Head & Neck, Endocrine Oncology, Moffitt Cancer Center, Tampa, FL, United States; 2 Department of Medical Oncology, Banner MD Anderson Cancer Center, Gilbert, AZ, United States; 3 Department of Thoracic-Head and Neck Medical Oncology, University of Texas M.D. Anderson Cancer Center, Houston, TX, United States; 4 Department of Internal Medicine, The University of Texas at Tyler, Tyler, TX, United States; 5 Center for Head & Neck Oncology, Dana-Farber Cancer Institute and Brigham and Women’s Hospital, Harvard Medical School, Boston, MA, United States; 6 Clinical Research Division, Fred Hutchinson Cancer Research Center, Seattle, WA, United States; 7 The David and Etta Jonas Center for Cellular Therapy, University of Chicago, Chicago, IL, United States; 8 Department of Medicine, University of California, Los Angeles, Los Angeles, CA, United States; 9 Kite Pharma Inc., Santa Monica, CA, United States; 10 Cell Therapy Service and the Center for Cell Engineering, Memorial Sloan Kettering Cancer Center, New York, NY, United States

**Keywords:** adoptive cell therapy (ACT), cell surface receptor drug targets, clinical trial results, immunotherapy, T-cell receptor (TCR)

## Abstract

Patients with relapsed/refractory (r/r) HPV-associated epithelial cancers have a poor prognosis. Engineered T cells expressing a T cell receptor (TCR) specific for HPV16 E7 can induce tumor regression. We conducted a Phase 1 trial of KITE-439, an investigational autologous T-cell product expressing TCR specific for HPV16 E7 in patients with r/r HPV16+ epithelial cancers. CD4+ and CD8+ T cells selected from leukapheresed peripheral blood mononuclear cells were stimulated with anti-CD3/anti-CD28 antibodies followed by retroviral transduction and expansion in the presence of interleukin-7/15 and an AKT inhibitor. Patients received lymphodepleting chemotherapy (cyclophosphamide 30 mg/kg/day for 2 days and fludarabine 25 mg/m^2^/day for 5 days) followed by a single infusion of KITE-439 with daily IL-2 (2.5×10^5^ IU/kg, up to 7 doses). The primary objectives were safety, tolerability, and efficacy; the primary endpoint was dose-limiting toxicities (DLTs). Eight HLA-A*02:01+ patients received KITE-439 (1×10^6^–1×10^8^ cells/kg). No DLTs occurred during the trial. In all patients, KITE-439 cells were detected in peripheral blood within 7 days post-infusion. Three patients experienced Grade 1–2 cytokine release syndrome related to KITE-439 (resolved within 1–5 days) and 4 had KITE-439–related Grade 1–2 neurologic events (resolved within a day). One patient achieved a partial response (from Day 35 to Month 3) and 7 had a best response of stable disease. These results suggest that KITE-439 has an acceptable safety profile for the treatment of patients with HPV-associated epithelial cancers. Further studies are needed to determine optimal manufacturing conditions and T-cell characteristics required for enhanced antitumor activity.

## Introduction

Globally, 5.5% of all cancers (690,000 new cases per year) are caused by the human papillomavirus (HPV) (1, 2). HPV16, a prominent HPV subtype, accounts for >80% of HPV+ head and neck squamous cell carcinoma (HNSCC), and 70% of HPV+ cervical cancers are attributable to HPV16 and another prominent subtype, HPV18 (3, 4).

Front-line therapy for these HPV-associated epithelial cancers includes surgery, radiation, chemotherapy, or chemoradiation therapy. Patients with metastatic or relapsed/refractory (r/r) HPV-associated epithelial cancers (e.g., HNSCC, cervical, and anogenital) may be treated with cisplatin plus concurrent radiotherapy, cisplatin-based combination regimens, or immunotherapy (5–10).

Patients with r/r or metastatic HPV-associated epithelial cancers have poor outcomes after experiencing disease progression following first-line chemotherapy, including with platinum-based regimens (5, 11). Overall, prognosis of r/r or metastatic HNSCC is poor with a median overall survival (OS) of 10–15 months (12), although patients with HPV-positive HNSCC have better prognosis compared with those who have HPV-negative disease even in the r/r or metastatic setting (12). Similarly, poor prognosis is observed in patients with advanced or r/r or metastatic cervical cancer who have progressed despite prior systemic therapy with median OS of 9–17 months (7, 13, 14). Given the poor overall clinical outcomes for patients with persistent, r/r, or metastatic HPV+ tumors, effective therapies are lacking and represent a critical unmet need.

HPV-driven cancers constitutively express the E7 oncoprotein, a conserved viral protein that plays an important role in carcinogenesis by inducing uncontrolled cellular proliferation through disruption of retinoblastoma protein function (15–17). Given that E7 is not expressed in healthy tissues, it is an attractive therapeutic target for these malignancies (18). E7 (11–19) is a human leukocyte antigen (HLA) class I-restricted epitope that is recognized in the context of HLA-A*02:01 (HLA-A2) (19). *HLA-A2* is the most common HLA class I allele in the US and is expressed by approximately 35%–50% of individuals across ethnic populations (20–23). T cells genetically engineered with a T-cell receptor (TCR) specific for HPV16 E7 were found to efficiently recognize and kill HPV16+ cervical and oropharyngeal cancer cell lines and mediate regression of HPV16+ tumors in a murine model (24). A National Cancer Institute (NCI) single-center Phase 1 clinical trial evaluated genetically engineered T cells with an E7 TCR for the treatment of patients with metastatic HPV16+ epithelial cancers. The treatment was well tolerated and showed evidence of clinical antitumor activity in refractory HPV16+ cancers (25).

KITE-439, an investigational autologous CD4+ and CD8+ engineered T-cell product using the NCI E7 TCR was developed based on the preliminary results from the aforementioned Phase 1 study. Engineered T-cell products that predominantly express markers of less differentiated T cells (i.e., T stem cell-like memory and central memory T cells) have been associated with improved clinical outcomes in patients with hematologic malignancies compared with more terminally differentiated T cells (26–29). To produce KITE-439, we augmented the NCI manufacturing process to include expansion of E7 TCR-transduced T cells with interleukin (IL)-7, IL-15, and an allosteric AKT-1/2 inhibitor to promote a ‘juvenile’ phenotype in KITE-439 cells consistent with a lower degree of differentiation (27). Here, we report results from the Phase 1 clinical trial of KITE-439 cells in patients with r/r HPV16+ epithelial cancers.

## Materials and methods

### Study design and patients

This was a Phase 1, open-label, multicenter study in adult patients with r/r HPV16+ cancers. Key eligibility criteria included advanced cancer defined as r/r disease after ≥1 line of therapy that included chemotherapy and disease that was not suitable for definitive locoregional therapy. Patients were confirmed to have HPV16+ tumors, HLA-A*02:01+ per local assessment, and had ≥1 measurable lesion per modified Response Evaluation Criteria in Solid Tumors (mRECIST) version 1.1 (30), confirmed by computed tomography (CT) or magnetic resonance imaging (MRI) performed after the last line of anticancer therapy and within 28 days before enrollment. Patients who received prior T-cell therapies, including those that target HPV, were excluded. Full eligibility criteria are provided in **Supplementary Materials and Methods**, **Supplementary Digital Content 1**.

The initial study design included Phase 1A (dose escalation) and 1B (dose expansion). During Phase 1A, a single-patient dose-escalation scheme was used for Cohorts 1–4 followed by a 3 + 3 design for Cohorts 5 and 6 (**Supplementary Figure 1**; **Supplementary Digital Content 2**). The following target dose levels of KITE-439 were assessed: Cohort 1, 1×10^6^ cells/kg; Cohort 2, 3×10^6^ cells/kg; Cohort 3, 1×10^7^ cells/kg; Cohort 4, 3×10^7^ cells/kg; Cohorts 5 and 6, 1×10^8^ cells/kg. Although the weight-based dose of KITE-439 cells was the same in Cohorts 5 and 6, the maximum allowable total number of KITE-439 cells was 5×10^9^ cells (± 20%) in Cohort 5 and 1×10^10^ cells in Cohort 6.

Dose-limiting toxicity (DLTs) were defined as any KITE-439–related Grade 3 toxicities starting within 21 days after KITE-439 infusion that did not resolve to Grade ≤2 within 48 hours, and any KITE-439–related Grade ≥4 toxicities starting within 21 days after KITE-439 infusion regardless of duration. During modified dose escalation, Cohorts 1–4 were single-patient cohorts unless the patient experienced a DLT. For Cohort 5, escalation would proceed to Cohort 6 if no DLTs occurred in the first 3 patients; if a DLT occurred in the first 3 patients in Cohorts 5 or 6 then an additional 3 patients would be enrolled in that cohort (see **Supplementary Materials and Methods**, **Supplementary Digital Content 1**, for additional information on DLT criteria, dose escalation criteria, and maximum tolerated dose [MTD]).

This study was conducted under a US Investigational New Drug application in accordance with recognized international scientific and ethical standards, including the International Council for Harmonisation of Technical Requirements for Pharmaceuticals for Human Use guideline for Good Clinical Practice and the original principles embodied in the Declaration of Helsinki. Screening to determine eligibility for enrollment was a 2-step process (pre-screening and screening), and patients were required to sign a separate informed consent for each step.

### Treatments and procedures

Peripheral blood mononuclear cells (PBMCs) were collected by leukapheresis and CD4+ and CD8+ T cells were selected from the apheresis product and stimulated with anti-CD3 and anti-CD28 antibodies. T cells were then transduced with a retroviral vector containing the HPV E7 TCR gene construct and expanded in culture medium supplemented with IL-7, IL-15, and an allosteric AKT-1/2 inhibitor (27, 31). KITE-439 cells were cryopreserved and remained frozen until the patient was ready for infusion.

Optional bridging therapy after leukapheresis and prior to lymphodepleting chemotherapy was to be administered at the investigator’s discretion. Patients received cyclophosphamide (30 mg/kg/day) for 2 days (reduced from 60 mg/kg per initial protocol amendment) and fludarabine (25 mg/m^2^/day) for 5 days as lymphodepleting chemotherapy before the administration KITE-439. Patients were hospitalized for at least 7 days for the infusion of KITE-439 and subsequent subcutaneous administration of IL-2 (2.5×10^5^ IU/kg once daily) on Days 0–6 of the inpatient hospitalization period for a maximum of 7 doses. Additionally, patients could have received treatment with granulocyte colony-stimulating factor (G-CSF) per institutional guidelines.

Clinical biospecimens, including tumor tissue, blood, and blood-derived samples were sent from clinical study centers to a central laboratory for sample processing and distribution to specialty laboratories or the sponsor for analysis.

### Endpoints and assessments

The primary objectives of this study were to evaluate the overall safety of KITE-439, determine a recommended Phase 1B dose (Phase 1A), and evaluate the efficacy of KITE-439 as measured by objective response rate (ORR) in Phase 1B. Secondary objectives included assessing safety and additional efficacy endpoints.

Safety endpoints included incidence of DLTs and adverse events (AEs) of special interest (AESI), e.g., cytokine release syndrome (CRS) and neurologic events (NEs). All AEs were coded using the Medical Dictionary for Regulatory Activities (MedDRA; version 24.1) and severity was graded using NCI Common Terminology Criteria for Adverse Events (CTCAE, version 5.0). AEs and serious AEs were reported from enrollment through 3 months after KITE-439 infusion. Severity of CRS events at the syndrome level were reported using a modification of the Lee grading scale (32).

CT or MRI scan was to be performed at screening, prior to lymphodepleting chemotherapy, at 4 weeks following KITE-439 infusion, and then every 3 months through Month 24.

Efficacy endpoints included ORR, progression-free survival (PFS), and OS. ORR was defined as the proportion of patients with either a complete response (CR) or partial response (PR) while on study, with investigator-assessed disease status (CR, PR, stable disease [SD], progressive disease [PD]) per mRECIST version 1.1 (30). Confirmation of CR or PR by radiological examination was required. PFS was defined as time from KITE-439 infusion to date of disease progression or death from any cause, and OS was defined as time from KITE-439 cell infusion to date of death due to any cause.

Pharmacokinetic (PK) assessments included monitoring of levels of KITE-439 TCR–expressing T cells in blood over time using flow cytometry. Pharmacodynamic (PD) assessments included monitoring levels of cytokines in serum over time after infusion, and in pleural fluid or cerebrospinal fluid on an *ad hoc* basis from patients who developed Grade ≥2 NEs. PK and PD assessments and T-cell phenotyping analyses are described in the **Supplementary Materials and Methods**, **Supplementary Digital Content 1**. Tumor sample analysis was performed to assess tumor genomics and cancer gene expression.

### Statistical analysis

The clinical hypothesis was that KITE-439 cells would have clinically meaningful antitumor activity as measured by ORR in patients with r/r HPV16+ cancers. Data were reported for the safety population which included all patients treated with any dose of KITE-439. Due to the small sample size, median PFS and median OS were estimated using descriptive statistics, and no formal statistical testing was performed.

### Data availability

Kite is committed to sharing clinical data with external medical experts and scientific researchers in the interest of advancing public health, and access can be requested by contacting medinfo@kitepharma.com.

## Results

### Patients

A total of 206 patients were prescreened for eligibility, of whom 109 (53%) did not meet screening criteria as they were HLA-A*02:01 negative, and 20 patients (10%) were ineligible as they were HPV16 negative (**Supplementary Figure 2**; **Supplementary Digital Content 3**). Of the 77 remaining eligible patients, 8 were enrolled at 4 sites in the US from June 19, 2019, to June 21, 2021 (eligible patients were enrolled one at a time per the dose escalation schematic). Six dose levels were explored as part of the planned dose escalation; the study did not proceed into Phase 1B due to sponsor decision to end the study early. As of the data cutoff date for this study (February 14, 2022), all patients had the opportunity to be followed for at least 6.5 months after infusion of KITE-439.

All patients (n=8) were white, 5 (63%) were male; all 8 patients had Stage IV disease (3 with unresectable locoregional disease; 5 with distant metastasis) with involvement of 1–4 anatomical sites, and Eastern Cooperative Oncology Group performance status of 0 (n=3, 38%) or 1 (n=5, 63%; **Table 1**). Most patients (n=7, 88%) had received ≥3 prior therapies (**Table 1**); 5 patients (3 with HNSCC, 1 with cervical cancer, and 1 with anogenital cancer) had prior treatment with pembrolizumab and 5 patients (4 with HNSCC and 1 with cervical cancer) had received prior treatment with nivolumab. Five patients (63%) had primary disease of HNSCC, 2 patients (25%) had cervical cancer, and 1 patient (13%) had anogenital cancer.

**Table 1.** Baseline characteristics.

Characteristic	All patients N=8
Median age, years (range)	59 (40–77)
<65 years, n (%)	6 (75)
≥65 years, n (%)	2 (25)
Male, n (%)	5 (63)
Ethnicity, n (%)	
Not Hispanic or Latino	8 (100)
Race, n (%)	
White	8 (100)
ECOG performance status, n (%)	
0	3 (38)
1	5 (63)
Disease characteristics, n (%)	
Stage IV	8 (100)
Locoregional	2 (25)
Distant metastasis	6 (75)
Primary tumor site	
Head and neck	5 (63)
Cervix	2 (25)
Anogenital	1 (13)
Metastatic sites^a^	
Lymph nodes	4 (50)
Lung/pleura	2 (25)
Liver	2 (25)
Bone	2 (25)
Abdomen	1 (13)
Median baseline tumor burden, mm (range)	84.5 (17–195)
≥3 prior therapies, n (%)^b^	7 (88)

^a^Exceeds 100% due to some patients having tumors at multiple sites.

^b^5 patients had prior treatment with pembrolizumab and 4 had prior treatment with nivolumab. ECOG, Eastern Cooperative Oncology Group.

All 8 patients received KITE-439. Dose-escalation Cohorts 1, 2, 3, 4, and 6 enrolled 1 patient each; Cohort 5 enrolled 3 patients. All patients received ≥1 dose of IL-2 following infusion of KITE-439 cells; 7 patients (88%) received <7 doses (due to IL-2 related AEs). No patients received bridging therapy during the interval between leukapheresis and the start of lymphodepleting chemotherapy. Two patients received cyclophosphamide dose levels of 60 mg/kg/day before lymphodepletion dosage was changed to 30 mg/kg/day (n=6) per a protocol amendment. Median time from leukapheresis to infusion was 30.5 days (range, 27–41 days). Manufacturing time for KITE-439 cells was 6–10 days. Median follow-up (time from infusion to death date or last date known alive) was 6.8 months (range, 1.8–16.4 months).

### Safety

No DLTs with KITE-439 occurred and no other significant dose-related increases in toxicity were reported. Across the dose levels studied (1×10^6^ to 1×10^8^ cells/kg), safety was comparable, and no maximum-tolerated dose was established. Seven patients (88%) had ≥1 AE that was Grade ≥3. Grade ≥3 AEs reported in ≥2 patients were decreased neutrophil count (n=6, 75%), anemia (n=5, 63%), decreased platelet count (n=5, 63%), decreased white blood cell count (n=3, 38%), diarrhea (n=2, 25%), and maculopapular rash (n=2, 25%; **Table 2**). Hematological cell counts were suppressed as expected due to lymphodepleting therapy; all patients recovered their cell counts except 1 who had prolonged cytopenia (on or after Day 30).

**Table 2.** Grade ≥3 treatment-emergent AEs by worst grade (N=8).

AE, n (%)	Any Worst Grade ≥3	Worst Grade 3	Worst Grade 4	Worst Grade 5	
Any Grade ≥3	7 (88)	0	5 (63)	2 (25)^a^	
Anemia	5 (63)	5 (63)	0	0	
Neutrophil count decreased	6 (75)	2 (25)	4 (50)	0	
Platelet count decreased	5 (63)	1 (13)	4 (50)	0	
WBC count decreased	3 (38)	1 (13)	2 (25)	0	
Diarrhea	2 (25)	2 (25)	0	0	
Maculopapular rash	2 (25)	2 (25)	0	0	
Leukocytosis	1 (13)	1 (13)	0	0	
Thrombocytopenia	1 (13)	0	1 (13)	0	
Lymphocyte count decreased	1 (13)	0	1 (13)	0	
Decreased appetite	1 (13)	0	0	1 (13)	
Hypocalcemia	1 (13)	1 (13)	0	0	
Hypophosphatemia	1 (13)	1 (13)	0	0	
Atrial flutter	1 (13)	1 (13)	0	0	
Non-cardiac chest pain	1 (13)	1 (13)	0	0	
Cellulitis	1 (13)	1 (13)	0	0	
Pneumonia	1 (13)	1 (13)	0	0	
Muscular weakness	1 (13)	1 (13)	0	0	
Squamous cell carcinoma	1 (13)	0	0	1 (13)^a^	
Syncope	1 (13)	1 (13)	0	0	
Hypoxia	1 (13)	1 (13)	0	0	
Hypotension	1 (13)	1 (13)	0	0	

^a^Includes 1 patient reported to have a Grade 5 AE (serious AE) of squamous cell carcinoma, although the investigator deemed that the patient’s primary cause of death was due to disease progression.

AE, adverse event; WBC, white blood cell.

Treatment-related AEs (TRAEs related to KITE-439) by preferred term and worst grade are provided in **Supplementary Table 1**, **Supplementary Digital Content 4**. Six patients (75%) had a TRAE. TRAEs that occurred in ≥2 patients were pyrexia (n=3, 38%), chills (n=2, 25%), and fatigue (n=2, 25%); all were Grade 1–2. One patient (13%) experienced Grade 3 hypotension related to KITE-439. There were no treatment-related deaths.

AESIs by preferred term and worst grade are provided in **Supplementary Table 2**, **Supplementary Digital Content 5**. No patients had Grade ≥3 CRS or Grade ≥3 NEs. Three patients (38%) had Grade 1–2 CRS, including the patient who had treatment-related Grade 3 hypotension; all events occurred on Day 0 or 1 and resolved within 1–5 days with no intervention. Four patients (50%) had ≥1 NE; 7 NEs were reported overall, and all were Grade 1–2. One patient (13%) had NEs related to KITE-439 that resolved within a day; NEs that occurred in the remaining 3 patients were deemed unrelated to KITE-439.

No patients died within 30 days of KITE-439 infusion. As of the data cutoff date, 7 of 8 patients (88%) had died: 6 from PD and 1 from an AE of decreased appetite reported 55 days after KITE-439 infusion (unrelated to KITE-439 treatment).

### Efficacy

**Figure 1:**
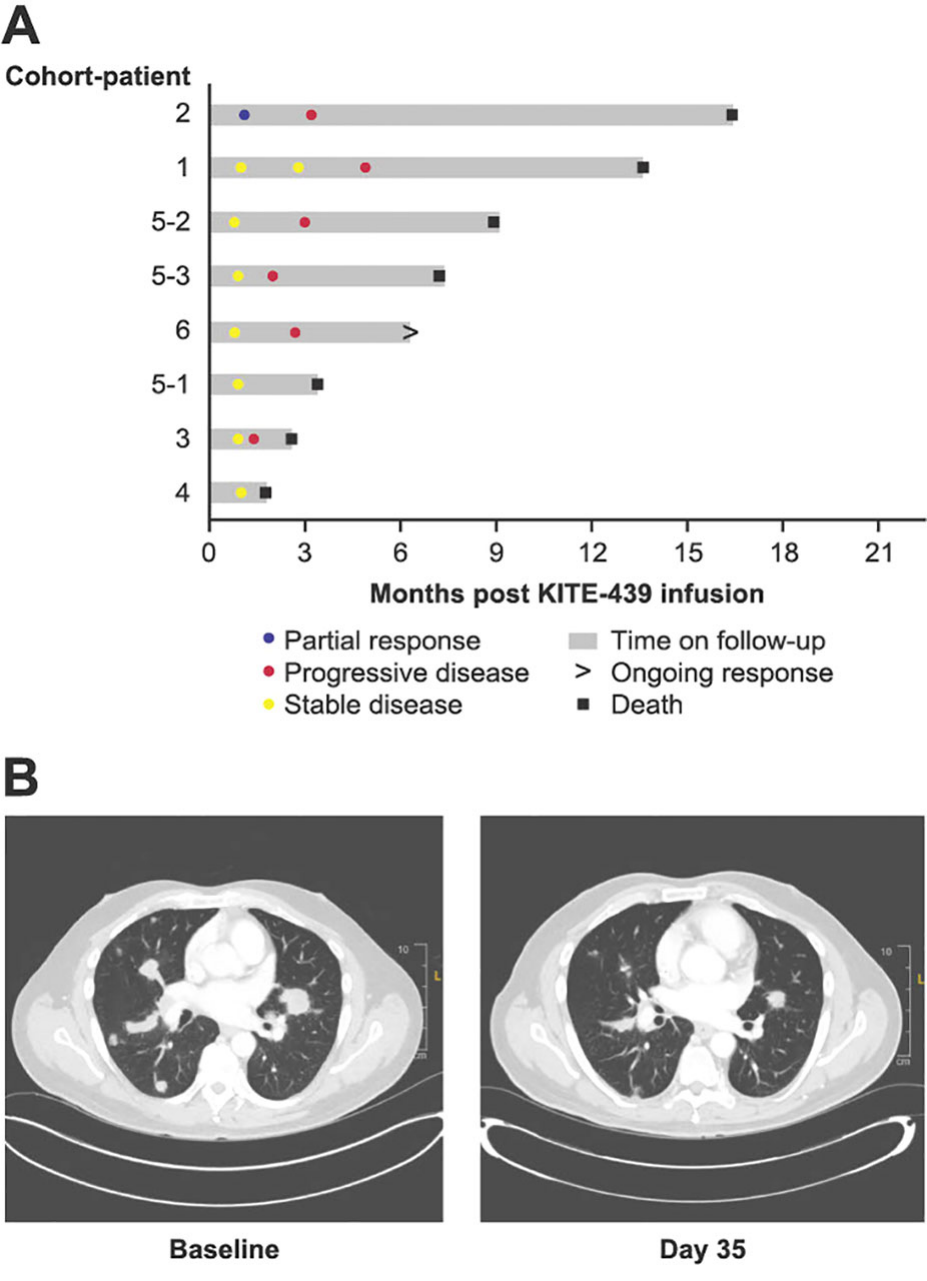
KITE-439 efficacy results. **(A)** Swimlane plot showing patient responses and survival status across all cohorts (n=8). **(B)** Representative contrast-enhanced chest CT images from a patient with metastatic HNSCC and pulmonary lesions at baseline (left) that showed a PR based on 42% tumor regression at Day 35 after KITE-439 administration (right). CT, computed tomography; HNSCC, head and neck squamous cell carcinoma; PR, partial response.

Among the 8 patients treated with KITE-439, 1 patient (13%) who was infused with 3×10^6^ KITE-439 cells (Cohort 2) had a best response of PR at study Day 35 followed by PD at the 3-month follow-up visit (**Figure 1**). The other 7 patients (88%; in Cohorts 1, 3, 4, 5, and 6) had a best response of SD at the Week 4 follow-up visit.

Six of 8 patients (75%; in Cohorts 1, 2, 5, and 6) were alive at the 3-month follow-up visit. As of the data cutoff date, 1 patient (13%) remained alive. The median PFS was 2.4 months (range, 0.9–4.9), and median OS was 7.4 months (range, 1.8–16.4). After the data cutoff date, the remaining patient died of PD after the 6-month follow-up assessment.

### Translational findings

#### Product immunophenotyping

**Figure 2:**
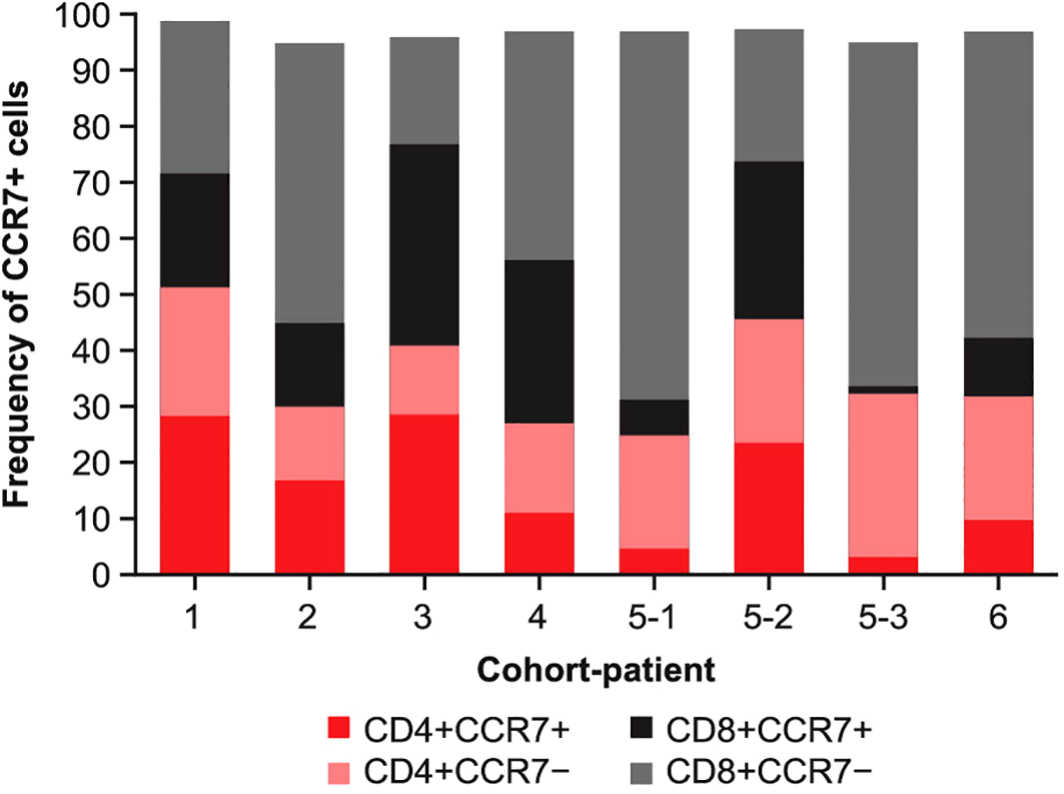
Phenotype distribution of T cells in KITE-439 products. Bar graph showing overall (transduced and non-transduced) frequencies of CD4+CCR7+, CD4+CCR7−, CD8+CCR7+, and CD8+CCR7− T cells in final KITE-439 infusion products across all patient cohorts, with predominance of CD8+CCR7− effector memory cells.

Transduction efficiency rates were 33%–79% for Cohorts 1–6, and most KITE-439 infusion products predominantly contained CD8+ T cells (**Figure 2**). Seven patients had KITE-439 products with overall CD4+/CD8+ T-cell ratios <1 (range, 0.35–0.88); 1 patient had a CD4+/CD8+ ratio of 1.08 (Cohort 1). The respective median frequencies of CD4+ and CD8+ T cells in apheresis material were 63.9% and 27.6% compared with 32.1% and 63.9% in KITE-439 products (**Supplementary Table 3**; **Supplementary Digital Content 6**). T-cell phenotypes in apheresis material (i.e., prior to lymphodepletion) were primarily CD4+CCR7+ cells (median frequency: 39.9% of CD3+ T cells), whereas infusion products were primarily CD8+CCR7− cells (median: 45.4% of CD3+ T cells; **Supplementary Figure 3**; **Supplementary Digital Content 7**). Patients in Cohorts 5 and 6 had lower frequencies of CCR7+ KITE-439 cells relative to other dose cohorts (**Figure 2**). Overall, the predominant infusion KITE-439 phenotype (CD8+CCR7−) was concordant with the phenotype of PBMCs through 4 weeks after infusion across dose levels (**Supplementary Figure 4**; **Supplementary Digital Content 8**).

**Figure 3:**
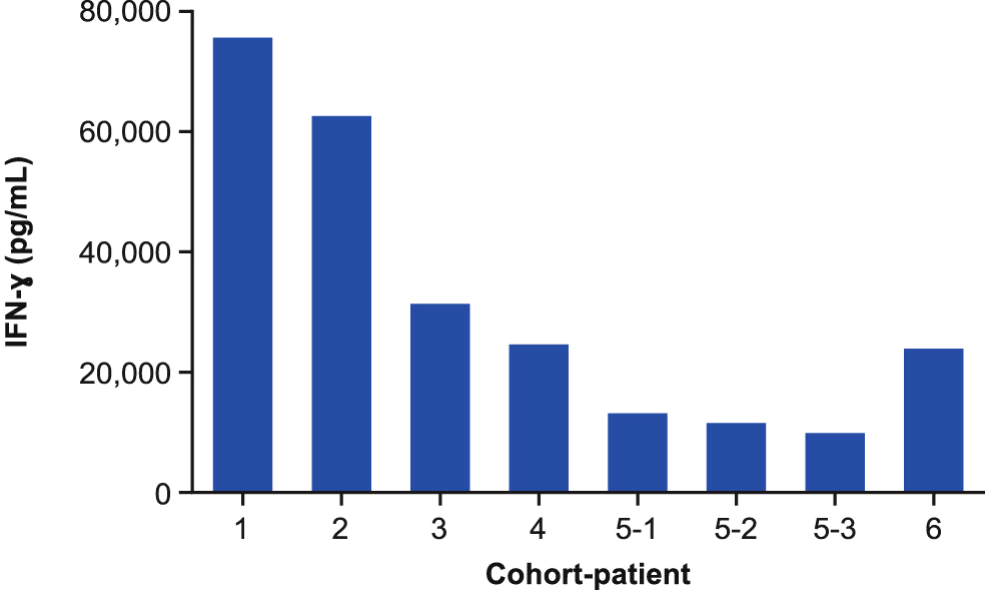
IFN-γ production by pre-infusion KITE-439 products. IFN-γ production (pg/mL) by KITE-439 cells following co-culture with HPV16 E7 peptide-loaded target cells, normalized to transduction rate across all patients. HPV, human papilloma virus; IFN-γ, interferon gamma.

All KITE-439 products showed ex vivo reactivity against HPV16 E7 peptide-presenting target cells. KITE-439 cells were co-cultured with peptide antigen-loaded cells and analyzed for interferon (IFN)-γ production (normalized to transduction rate). Products in the highest dose-level cohorts (Cohorts 5 and 6) had lower capacity for IFN-γ production compared with the lower dose levels (Cohorts 1–4; **Figure 3**).

#### Clinical pharmacology

**Figure 4:**
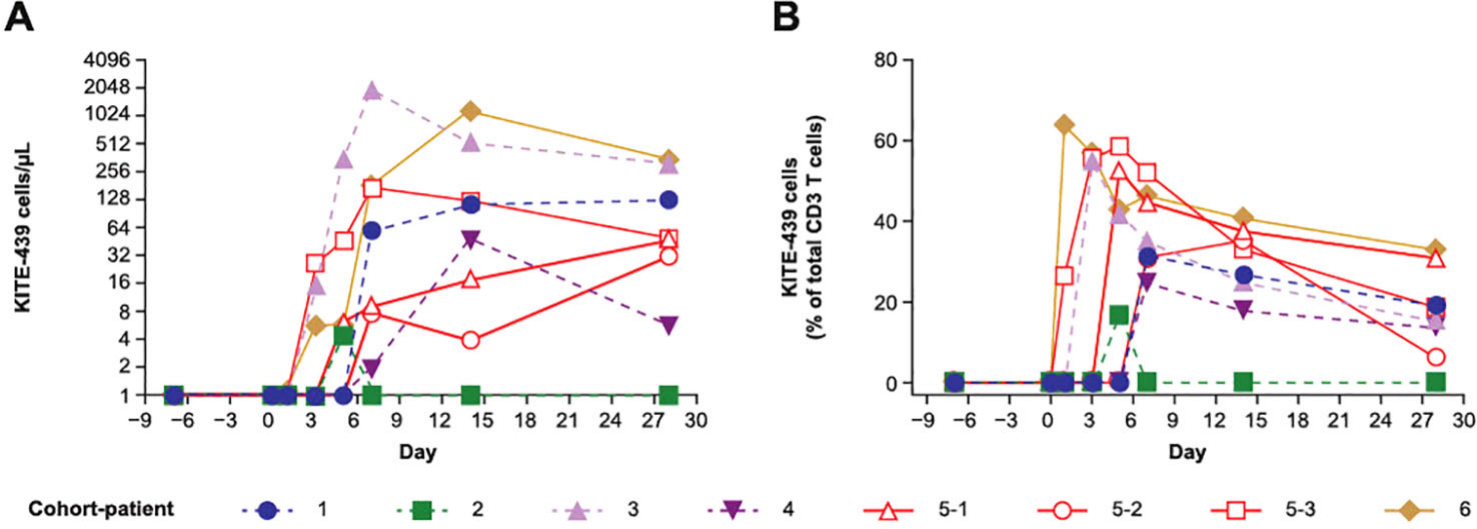
KITE-439 T-cell expansion and persistence in peripheral blood. **(A)** Concentration (cells/μL) and **(B)** proportion (% of CD3+ T cells) of KITE-439 cells following infusion through Day 30 for all patients.

KITE-439 cells were detected in peripheral blood within the first 7 days after infusion in all patients as assessed by the number of cells (per μL of blood; **Figure 4A**) and by frequency (as a percentage of total CD3+ T cells, **Figure 4B**). Median peak level of KITE-439 cells was 88.21 cells/μL (range, 4.54–1,955.48 cells/μL), and median time to peak was 14 days after KITE-439 infusion (range, 6–29 days). On Week 4 after infusion, the median number of cells was 48.18 cells/μL (range, 0.00–346.67 cells/μL); 1 patient who had a partial response had no detectable KITE-439 cells in the peripheral blood at Week 4.

The number of KITE-439 cells observed in peripheral blood was not associated with response to treatment in this small patient population. However, the frequency of KITE-439 cells in the CD3+ T-cell population was concordant with dose; patients in Cohorts 5 and 6 who received a higher KITE-439 dose level tended to have a higher frequency of KITE-439 cells in peripheral blood through Day 15 after infusion. One patient (Cohort 4) had pleural fluid collected 2 weeks after infusion and measurement of KITE-439 cells showed a similar frequency between peripheral blood (15.7%) and pleural fluid (13.9%; **Supplementary Figure 5**; **Supplementary Digital Content 9**). Following infusion, this patient had a marked reduction in pleural effusion drainage requirements, consistent with antitumor activity of KITE-439.

**Figure 5:**
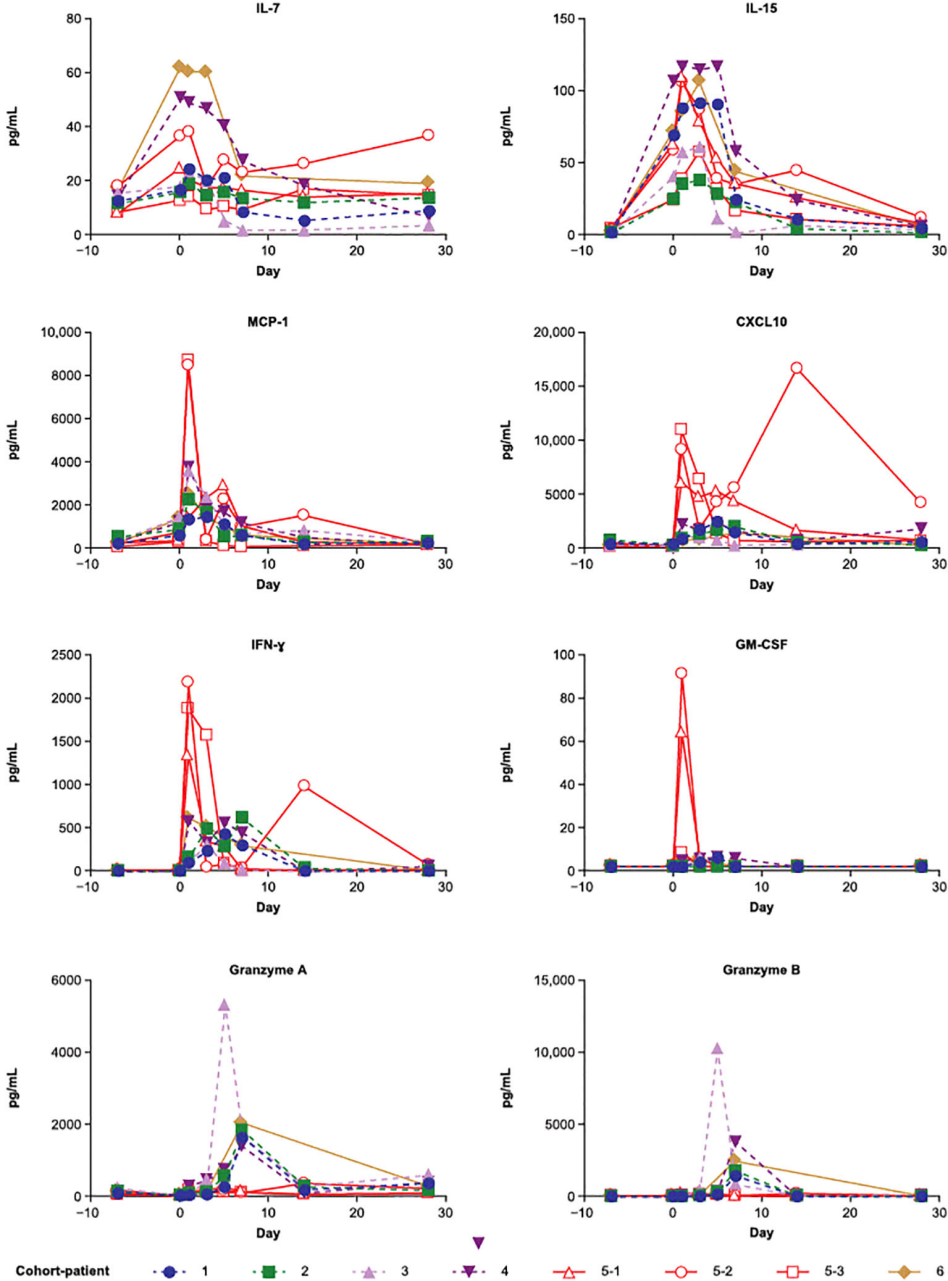
Serum cytokine and chemokine profiles following KITE-439 infusion. Time course of IL-7, IL-15, MCP-1, CXCL10, IFN-γ, GM-CSF, Granzyme A, and Granzyme B concentrations (pg/mL) across all patient cohorts from pre-infusion to Day 30. CXCL10, C-X-C motif chemokine ligand 10; GM-CSF, granulocyte-macrophage colony-stimulating factor; IFN-γ, interferon gamma; IL, interleukin; MCP-1, monocyte chemoattractant protein-1.

Measurement of key serum analytes after KITE-439 infusion showed that median peak levels of homeostatic (IL-15), inflammatory and immune-modulating cytokines (IFN-γ, granulocyte-macrophage colony-stimulating factor), chemokines (e.g., monocyte chemoattractant protein-1 [MCP-1], C-X-C motif chemokine ligand 10 [CXCL10]), and T-cell effector proteins were increased 2-fold or more from baseline (**Figure 5**). Median times to peak serum analyte levels were 2–8 days. IL-7, IL-15, and MCP-1 levels were increasing prior to infusion of KITE-439 (after lymphodepleting chemotherapy).

#### Tumor genomics

Genomic analysis of pretreatment tumors was performed *post hoc* to identify pre-existing genetic defects in genes encoding molecules critical to T cell-mediated recognition of E7, including HLA-A*2:01 and *B2M* (the gene encoding β2 microglobulin). A pretreatment tumor sample from the patient in Cohort 3 showed a complete loss of both HLA-A*02:01 alleles (who also had the highest peak of KITE-439 cells); other patients did not show any mutation or loss of heterozygosity in their HLA-A*02:01 allele. Further, no mutations or loss of *B2M* was observed in any of the samples tested (**Supplementary Table 4**; **Supplementary Digital Content 10**).

HPV16 viral genome was detected in all tumor samples tested; however, we were not able to confirm that the E7 (11–19) epitope (recognized by KITE-439 cells) was present in these tumor samples.

## Discussion

Most patients with metastatic and/or r/r HPV-associated epithelial cancers do not achieve objective responses with currently available therapies and have a significant unmet need for effective treatment options (9). As a novel approach, we designed KITE-439 as an engineered TCR T-cell therapy that targets an epitope derived from HPV16 E7 that is commonly expressed in HPV16-positive tumors (3, 25). In the dose-escalation portion of this study, KITE-439 had a manageable safety profile comparable with CAR T-cell therapies (33, 34), and no DLTs occurred (primary endpoint) at any dose level. KITE-439–related CRS and NEs were low severity (Grade 1–2) and resolved within 1–5 days, supporting the safety of engineered TCR therapies in patients with r/r HPV-associated cancers.

Although small sample size limits the ability to form definitive conclusions, safety findings with KITE-439 are consistent with evidence to date suggesting that engineered TCR therapies may have some advantages compared with CAR T cells (35). In the case of KITE-439, product cells specifically target tumor antigen-presenting cells, thereby limiting off-tumor/on-target effects that can occur with some CAR T-cell products (18, 35). Upon recognition of the E7 tumor antigen, KITE-439 cells undergo activation with physiologic signaling and co-stimulation compared with the supraphysiologic T-cell activation that occurs with CAR T cells upon stimulation (35). Overall, the safety profile of KITE-439 is consistent with its proposed mechanism of action and the potential safety benefits of engineered TCR T cells (36).

The limited response rate observed with KITE-439 (a single patient with a PR who then had PD by Month 3) was in the context of a heavily pretreated patient population, as 7 of the 8 patients in the study had received 3 or more prior therapies. Multiple treatment lines, particularly those that include immune checkpoint inhibitors (7 patients had prior nivolumab or pembrolizumab), can alter the tumor microenvironment and lead to changes that may induce resistance to future therapies. Mechanisms for this may include development of an immunosuppressive state that inhibits T-cell function and that potentially impairs antigen presentation, or leads to depletion/exhaustion of CD4+ helper T cells or other immune cells crucial for the function and persistence of engineered TCR T cells (37–39). Extensive prior lines of therapy can also lead to epigenetic changes in the tumor microenvironment that may promote T-cell exhaustion and limit durability of function (38). Given the low efficacy observed, we could not make any conclusions about the effect of KITE-439 dose level on objective responses. The patient who had a PR received a lower dose than 6 of the patients with a best response of SD.

Based on absolute cell numbers in peripheral blood, expansion of KITE-439 cells after infusion was not consistent with the infusion dose across cohorts. Overall, patients in Cohorts 5 and 6 who received the highest doses had higher frequencies of KITE-439 cells as a proportion of CD3 T cells in peripheral blood at peak expansion. Although this study population was too small to make inferences associating KITE-439 cell expansion with efficacy, it is noteworthy that no patients in Cohort 5 or 6 had an objective response. In hematological malignancies, efficacy of adoptive T-cell therapies can be highly dependent on the degree of T-cell expansion and persistence after infusion, which are among factors that can drive variability in treatment response independent of the initial dose received (39–42). In the case of solid tumors in this study, adoptively transferred T cells must migrate to the tumor site to have a significant treatment effect, and correlations of PK measurements in the blood with efficacy are less meaningful. Notably, we did find that levels of KITE-439 cells in the pleural fluid of 1 patient with metastasis to the lung were comparable to that of peripheral blood. Although this patient did not achieve a response with KITE-439 (however, there was significant improvement in pleural fluid accumulation suggesting a possible clinical benefit), this observation demonstrates the feasibility of KITE-439 cells to traffic to a tumor-affected compartment. In the prior NCI study using the same E7 TCR–engineered T cells, there was prolonged persistence at an appreciable frequency for at least 100 days in some patients in high-dose cohorts, yet this persistence was not necessarily concordant with response (25). In a separate study investigating E6 TCR–engineered T cells, a patient who had a 6-month PR showed 26% peripheral blood engraftment of the therapeutic cells 17 months after treatment (43). In our trial, KITE-439 cells comprised 15%–35% of all circulating T cells through Day 28 in 75% of patients; however, 88% of patients had either died or had disease progression by their Month 3 visit. Taken together, these pharmacokinetic data indicate that the low efficacy with KITE-439 was neither related to lack of product expansion nor persistence (44, 45).

The prior NCI study of the same TCR construct used in KITE-439 enrolled 12 patients with metastatic HPV-associated epithelial cancers, which had similar baseline characteristics to patients in our study, including a similar extensive treatment history (3–7 prior therapies) (25). Patients in the NCI study achieved an ORR of 50%, based on 6 patients with a PR. Although patient populations shared key characteristics, differences in both study design (single- vs multicenter) and manufacturing processes may have contributed to the different objective response outcomes. Notably, transduction rates were considerably higher in the NCI study (93%–99%) versus with KITE-439 (33%–79%), which, among other differences in the ex vivo stimulation and expansion protocols, may have had downstream impacts on efficacy.

The phenotypic composition of infused engineered T cells can also influence treatment outcomes in patients with solid tumors (40, 45). KITE-439 was designed to improve upon the NCI product with modifications in the manufacturing process that were intended to produce T cells of a ‘juvenile’ phenotype consistent with a lower degree of differentiation. It was hypothesized that a lesser degree of T-cell differentiation would enhance antitumor activity based on increased proliferation and persistence compared with more differentiated, effector-like T cells (27, 46). Although effector-like T cells may have the potential for more immediate antitumor effects, they also exhibit reduced self-renewal ability and survival compared with less differentiated cells (40). Thus, the manufacturing of KITE-439 included stimulation of E7 TCR-transduced T cells with IL-7, IL-15, and an allosteric AKT-1/2 inhibitor, with the aim of skewing product differentiation status to a juvenile/central memory phenotype. Additionally, KITE-439 had a reduced manufacturing time of up to 10 days compared with approximately 23 days in the NCI study (inclusive of additional culture time in the presence of irradiated allogeneic PBMCs and IL-2 intended to improve yield) (25), which could have also favored less differentiation. Despite these design modifications for KITE-439, there was a shift from a predominant CD4+CCR7+ cell population in apheresis material to a CD8+CCR7− population in the infusion product. This shift was particularly evident at higher dose levels, in which patients had the lowest frequencies of undifferentiated (CCR7+) cells and the most rapid time to peak serum IFN-γ levels after infusion. In Cohorts 4–6, additional manufacturing time required to produce the higher- versus lower-dose KITE-439 products (10 vs 6 days) may have allowed more time for differentiation to occur, but nonetheless, these steps did not lead to increased antitumor activity as intended. Given the higher, acute cytolytic capacity of effector memory cells, it is possible that the stronger efficacy signal observed in the NCI study was a result of transduction efficiency combined with a high proportion of effector memory product cells. Further investigation will be required to fully understand how manufacturing variables such as T-cell phenotypes and transduction rates, combined with patient-specific factors, influence the effectiveness of engineered TCR T-cell therapies.

This study had several limitations, including the small sample size and early termination prior to fully exploring all dose levels. Comparisons of results obtained with the NCI E7-TCR and KITE-439 products should be made with caution based on small sample sizes in both studies. Challenges associated with precision therapies, such as narrow enrollment criteria based on HPV positivity and HLA typing, contributed to the KITE-439 study being stopped prior to fully exploring all dose levels. As expected, the different tumor types among patients in this study is a confounding factor for analyzing outcomes with KITE-439. Also, results obtained in studies of HPV-targeted therapies in patients with specific tumor types are not generalizable to all patients with HPV-associated epithelial cancers.

No DLTs were observed with KITE-439 suggesting that HPV-targeted TCR-engineered T-cell therapy may have an acceptable safety profile in patients with HPV-associated cancers. Additional investigation is needed to determine the optimal manufacturing conditions and T-cell phenotype/differentiation status best suited for antitumor activity of autologous engineered T cells for patients with epithelial tumors.

## Data Availability

The original contributions presented in the study are included in the article/**Supplementary Material**. Further inquiries can be directed to the corresponding author.
