# Supplementary material for: A phase 1 trial of HPV16 E7 T-cell receptor-engineered T cells in patients with relapsed/refractory HPV16-positive cancers (KITE-439 trial)
Source: Front Oncol. 2026 May 21;16:1809354. doi: 10.3389/fonc.2026.1809354 (PMC13244869; doi:10.3389/fonc.2026.1809354)
Supplement: Supplementary file 1 [file DataSheet1.docx]

**A Phase 1 Trial of HPV16 E7 T-cell Receptor-Engineered T Cells in Patients With Relapsed/Refractory HPV16-Positive Cancers (KITE-439 Trial)**

Kedar Kirtane, Jiaxin Niu, George Blumenschein, Jr., Erminia Massarelli,
Glenn J. Hanna, Sylvia Lee, Michael R. Bishop, Gottfried E. Konecny, Daqin Mao,
Yan Zheng, Katherine Rodriguez, Jenny J. Kim, Chad Williams, Colleen Schweitzer,
Sabina Adhikary, A. Scott Jung, Christopher A. Klebanoff

**SUPPLEMENTAL DIGITAL CONTENT**

Table of Contents

SUPPLEMENTAL MATERIALS AND METHODS 3

Inclusion criteria 3

Exclusion criteria 4

Dose-limiting toxicity (DLT) criteria 8

Dose escalation and maximum tolerated dose (MTD) criteria 9

T-cell phenotyping 12

PK assessments 12

IFNγ-release assay 13

Measurement of serum analytes 13

SUPPLEMENTAL TABLES 14

Table S1. TRAEs by worst grade (N=8) 14

Table S2. AESI by worst grade (N=8) 15

Table S3. CD4+ and CD8+ T cell frequency in apheresis versus KITE-439 products 16

Table S4. Analysis of *HLA-A*02:01* allele, *B2M*, and HPV oncogene status 17

SUPPLEMENTAL FIGURES 18

Figure S1. Study design 18

Figure S2. Patient disposition 19

Figure S3. Phenotypic evaluation of apheresis material and KITE-439 infusion products 20

Figure S4. T-cell phenotype in peripheral blood pre- and post-KITE-439 infusion 21

Figure S5. Frequency of KITE-439 cells in peripheral blood and pleural fluid in the Cohort 4 patient (lung metastasis) 22

SUPPLEMENTAL REFERENCES 23

# SUPPLEMENTAL MATERIALS AND METHODS

## Inclusion criteria

1. Age ≥18 years
2. Advanced cancer defined as relapsed or refractory disease after at least 1 line of therapy that included systemic chemotherapy and that was not amenable to definitive locoregional therapy
3. HPV16+ tumor as confirmed by the central laboratory
4. HLA type was *HLA-A*02.01*^+^ per local assessment
5. At least 1 measurable lesion per modified Response Evaluation Criteria in Solid Tumors (RECIST) version 1.1 per computed tomography (CT) or magnetic resonance imaging (MRI) performed after the last line of anticancer therapy and within 28 days before enrollment
6. No evidence of central nervous system (CNS) disease by MRI or CT (if MRI was not feasible) of the brain performed within 28 days before enrollment. Patients with prior brain metastasis, which had been treated with definitive therapy (surgical resection or radiation therapy), were eligible provided that the definitive therapy was completed more than 6 months before screening
7. Eastern Cooperative Oncology Group (ECOG) performance status 0 or 1
8. At least 2 weeks or 5 half-lives, whichever was shorter, must have elapsed since any prior systemic therapy at the time of enrollment (i.e., leukapheresis)
9. Toxicities due to prior therapy must have been recovered to baseline or ≤Grade 1, except for clinically nonsignificant toxicities, such as alopecia
10. Adequate bone marrow function (in absence of transfusion or growth factor support) as evidenced by:
11. Absolute neutrophil count (ANC) ≥1000/mm^3^
12. Platelet ≥100/mm^3^
13. Hemoglobin >8 g/dL
14. Adequate renal, hepatic, cardiac, and pulmonary function as evidenced by:
15. Creatinine clearance (as estimated by Cockcroft Gault) ≥60 cc/min (24-hour urine creatinine clearance was also acceptable)
16. Alanine aminotransferase/aspartate aminotransferase (ALT/AST) ≤2.5 × upper limit of normal (ULN) or ≤5 × ULN if documented liver metastases
17. Total bilirubin ≤1.5 mg/dL, except in patients with Gilbert’s Syndrome in whom total bilirubin must have been ≤3.0 mg/dL
18. Cardiac ejection fraction ≥50%, no evidence of pericardial effusion, as determined by an echocardiogram (ECHO), and no clinically significant electrocardiogram (ECG) findings (for ejection fraction only, multi-gated acquisition (MUGA) scan was also acceptable)
19. No clinically significant pleural effusion
20. Baseline oxygen saturation >92% on room air

## Exclusion criteria

1. History of malignancy other than non-melanoma skin cancer, carcinoma in situ (e.g., cervix, bladder, breast), or low-grade prostate cancer for which watch-and-wait approach is standard of care, unless disease-free for at least 3 years before enrollment
2. History of myocardial infarction, cardiac angioplasty or stenting, unstable angina, cardiac arrhythmia requiring antiarrhythmic or procedure, or other clinically significant cardiac disease within 12 months before enrollment
3. History of stroke or transient ischemic attack (TIA) within 12 months before enrollment
4. History of symptomatic deep vein thrombosis (DVT) or pulmonary embolism within 6 months before enrollment, catheter-associated thrombosis was not included as an exclusion criterion
5. Prior T-cell therapy, including those that target HPV; vaccines were not included as an exclusion criterion
6. Live vaccine ≤4 weeks before enrollment
7. Systemic corticosteroid therapy within 7 days before enrollment. Topical and inhaled corticosteroids in standard doses and physiologic replacement for patients with adrenal insufficiency were allowed. Any new doses ≥5 mg/day of prednisone or equivalent doses of other corticosteroids were not allowed
8. History of severe immediate hypersensitivity reaction to cyclophosphamide, fludarabine, or interleukin-2 (IL-2)
9. History of severe, immediate hypersensitivity reaction attributed to aminoglycosides
10. Presence of fungal, bacterial, viral, or other infection requiring anti-microbials for management. Simple urinary tract infection (UTI) and uncomplicated bacterial pharyngitis were permitted if responding to active treatment and after consultation with the Kite medical monitor
11. Presence of any indwelling line or drain (e.g., percutaneous nephrostomy tube, indwelling Foley catheter, biliary drain, or pleural/peritoneal/pericardial catheter). Dedicated central venous access catheters, such as a Port-a-Cath or Hickman catheter, as well as feeding tubes such as a gastrostomy tube were permitted
12. Primary immunodeficiency
13. History of autoimmune disease (e.g., Crohn’s, rheumatoid arthritis, systemic lupus) resulting in end organ injury or requiring systemic immunosuppression/systemic disease modifying agents within the last 2 years before enrollment**.** Immune-mediated toxicity due to immunotherapy for treatment of cancer was not exclusionary if a healthcare professional confirmed complete resolution while off systemic immunosuppressive/systemic disease modifying agents with no evidence of recurrence for at least 6 months before enrollment, and treatment with systemic immunosuppressive/systemic disease modifying agents, with the exception of physiologic steroid replacement, was not required during study participation
14. Known history of infection with human immunodeficiency virus (HIV), hepatitis B (hepatitis B surface antigen [HBsAg] positive), or hepatitis C (anti-HCV positive). A history of treated hepatitis B or hepatitis C was permitted if the viral load was undetectable per quantitative polymerase chain reaction (qPCR) and/or nucleic acid testing
15. Females who were pregnant as confirmed by a positive serum or urine pregnancy test or were breastfeeding. Females who had undergone surgical sterilization or who were postmenopausal for at least 2 years were not considered to be of childbearing potential
16. Males or females of childbearing potential who were not willing to practice birth control from the time of consent through 6 months after the completion of KITE-439
17. In the investigator’s judgment, if the patient was unlikely to complete all protocol-required study visits or procedures, including follow-up visits, or comply with the study requirements for participation
18. Known history of illicit psychostimulant use (cocaine, methamphetamine, MDMA, PCP) within 12 months before enrollment and/or positive urine toxicology during the screening phase

## Dose-limiting toxicity (DLT) criteria

DLTs were defined as all KITE-439–related Grade 3 toxicities that started within the first 21 days after the KITE-439 infusion and that did not resolve to Grade ≤2 within 48 hours. All KITE-439–related Grade ≥4 toxicities that started within the first 21 days after the KITE-439 infusion, regardless of duration, were also considered a DLT.

Exceptions were:

- Myelosuppression (includes bleeding in the setting of platelet count less than 50 x 10^9^/L and documented bacterial infections in the setting of neutropenia), defined as lymphopenia, neutropenia, decreased hemoglobin, and thrombocytopenia (because these are expected due to the chemotherapy preparative regimen)
- Immediate hypersensitivity reactions (excluding symptomatic bronchospasm and Grade 4 hypotension) occurring within 2 hours of cell infusion that are reversible to Grade ≤2 within 24 hours of cell administration with standard therapy
- Grade 3 or Grade 4 fever
- Tumor lysis syndrome (TLS), including associated manifestations attributable to TLS (e.g., electrolyte abnormalities, renal function, hyperuricemia) Grade 3 transaminase, alkaline phosphatase, bilirubin, or other liver function test elevation, provided there is resolution to Grade ≤2 within 14 days
- Grade 4 transient serum hepatic enzyme abnormalities, provided there is resolution to Grade ≤3 within <72 hours and Grade ≤2 within 14 days
- Grade 3 CRS (Lee 2014 criteria^1^) must resolve to Grade ≤2 within 72 hours
- AEs that are related to lymphodepleting chemotherapy, IL-2, or disease progression

## Dose escalation and maximum tolerated dose (MTD) criteria

During Phase 1A, the study will employ a single-patient dose escalation scheme for the first 4 of the planned 6 dose cohorts. Patients will be monitored for the occurrence of DLTs within the first 21 days after receiving the KITE-439 infusion. If the patient does not experience a DLT during the DLT window, then the next patient will be dosed at the next higher dose cohort for the first 4 of the planned 6 dose cohorts. Once the highest dose cohort is reached, it will follow a 3+3 design, (noted as Cohort 5 and 6 [n=3+3] below). If the patient does experience a DLT, then the rules as outlined below will apply.

Enrollment in the dose cohorts will proceed as follows based on the incidence of DLTs:

- If there are no DLTs in any dose cohort from Cohort 1 to Cohort 6, enrollment will proceed as follows:
  - Cohort 1 (n=1): 1×106 E7 TCR T cells/kg
  - Cohort 2 (n=1): 3×106 E7 TCR T cells/kg
  - Cohort 3 (n=1): 1×107 E7 TCR T cells/kg
  - Cohort 4 (n=1): 3×107 E7 TCR T cells/kg
  - Cohort 5 (n=3+3): 1×108 E7 TCR T cells/kg
  - Cohort 6^a^ (n=3+3): 1×10**8** E7 TCR T cells/kg

^a^For Cohort 6, the target dose is 1×108 E7 TCR T cells/kg and the total number of cells will be calculated based on weight. The minimally acceptable dose for patients above 60 kg will be 6×109 E7 TCR T cells. The maximum total number of cells in this cohort will be 1×1010 E7 TCR T cells.

In single-patient dose escalation/de-escalation design,

- If the initial patient had a DLT in Cohort 1, Cohort 1 will be expanded to 6 patients. If the incidence of DLT is <33% after the expansion, then enrollment will continue to the next dose cohort with all subsequent cohorts being expanded to 3+3. If there are ≥2 DLTs in Cohort 1, the study may explore a lower KITE-439 dose (Cohort −1).
- If the first patient in dose Cohort 2, 3, or 4 experiences a DLT, enrollment will proceed as follows:
  - The previous dose cohort will be expanded to 3+3. If there are <33% DLTs in the previous dose cohort after the expansion or if the previous dose cohort had already been expanded, then the current dose cohort will be expanded to 6 patients.
    - If there are <33% DLTs in the expanded cohort, then enrollment will continue to the next dose cohort.
    - All subsequent dose cohorts will be expanded to 3+3.
    - If the incidence of DLTs remains <33%, then enrollment will continue with this approach until the highest dose cohort is reached.

The highest dose cohort in which the incidence of DLTs is <33% will be designated the MTD.

- If there are ≥2 DLTs in any dose cohort after the first dose cohort, enrollment will proceed as follows:
  - The previous dose cohort will be expanded to 3+3, unless it was already expanded in which case the dose tested in the previous cohort will be designated the MTD.
    - If there is 0 or 1 DLT (i.e., 0 DLT in 3 patients or 1 DLT in 6 patients) in the previous cohort, then that dose cohort will be designated the MTD.
    - If there are ≥2 DLTs (i.e., ≥2 DLTs in 6 patients) in the previous cohort, then the cohort before the previous cohort will be expanded to 3+3 and so on.

The highest dose cohort in which the incidence of DLTs is <33% will be designated the MTD.

At any time, when ≥2 DLTs have been observed prior to completing dosing of the planned number of patients in a cohort, enrollment and dosing of additional patients in that cohort will be stopped, and the cohort will be deemed unsafe.

In 3+3 study design:

- If there is no DLT observed among the first 3 patients of Cohort 5, then the enrollment will continue to Cohort 6.
- If there is a DLT observed among the first 3 patients, then an additional 3 patients will be enrolled into the same cohort.
- If there is no DLT among the additional 3 patients in Cohort 5, then the enrollment will continue to Cohort 6.
- If there are ≥2 DLTs among the first 3 patients or ≥1 DLT among the additional 3 patients in Cohort 5, then follow the single-patient dose escalation/de-escalation process described in the “If there are ≥2 DLTs in any dose cohort after the first dose cohort” above.
- Cohort 6 will follow the same process as Cohort 5, except:
  - If there is no DLT in the first 3 patients or 1 DLT in the first 6 patients, Cohort 6 may be designated as MTD.
  - Otherwise Cohort 5 may be designated as MTD.

Based on the incidence of DLTs and other accumulating data, such as AEs and objective response outcomes, each disease type will be evaluated to proceed to the Phase 1B portion of the study. Kite/sponsor reserves the right to recommend 1 or more doses to be evaluated in Phase 1B, provided that each dose is at or below the MTD.

The decision to proceed with a specific dose or doses of KITE-439 in Phase 1B was made by Kite and formally communicated to participating sites in a separate communication.

## T-cell phenotyping

CCR7 expression among total CD4+ or CD8+ T cells within KITE-439 products, apheresis material, and patient PBMCs (collected before infusion and up to 4 weeks after infusion) was measured by flow cytometry using antibodies against CD3 (UCHT1; BD Biosciences), CD4 (SK3, BioLegend), CD8 (SK1, BD Biosciences), and CCR7. Cells were gated on live, singlet, lymphocytes.

## PK assessments

KITE-439 engineered T cell were measured by flow cytometry in the peripheral blood of patients after infusion as previously described.^2^ Briefly, HPV16 E7 TCR–positive T cells were detected using antibodies against CD3 (UCHT1; BD Biosciences), mouse TCR β-chain constant region (H57–595; eBioscience/Thermo Fisher Scientific), CD4 (SK3, BioLegend), and CD8 (SK1, BD Biosciences), an HLA-A*02:01-E7_11–19_ tetramer (peptide sequence YMLDLQPET, MBL), and a fixable viability dye (eBioscience/Thermo Fisher Scientific). Data were analyzed with FlowJo software (Becton Dickinson). E7 TCR T cells were measured as tetramer-bound cells that were positive for mouse TCR β-chain constant region within the CD3+ T-cell population gated on live, singlet, lymphocytes.

## IFNγ-release assay

Co-culture IFNγ-release assays were performed using E7 peptide–pulsed CEM-T2 cells mixed with E7 TCR–transduced KITE-439 product cells as previously described.^3^ IFN**γ** levels in cell culture supernatants following overnight incubation were measured using a qualified ELISA. Data were normalized by the transduction rate across all patients.

## Measurement of serum analytes

Soluble serum analytes were measured in samples collected before KITE-439 infusion and up to Day 30 after infusion using qualified immunoassay platforms as previously described.^3,4^ Analyte panels included in this study were MSD V-PLEX Plus Cytokine Panel 1 [Human] Kit (IL-7, IL-15, and GM-CSF), MSD V-PLEX Plus Chemokine Panel 1 (Human) Kit (MCP-1, CXCL10), MSD V-PLEX Plus Proinflammatory Panel 1 (Human) Kit (IFN**γ**), and ProteinSimple Simple Plex (granzyme A and granzyme B).

# SUPPLEMENTAL TABLES

| Table S1. TRAEs by worst grade (N=8) | | | | | | |
| --- | --- | --- | --- | --- | --- | --- |
| **n (%)** | **Any** | **Worst**  **Grade 1** | **Worst**  **Grade 2** | **Worst**  **Grade 3** | **Worst**  **Grade 4** | **Worst**  **Grade 5** |
| TRAE | 6 (75) | 1 (13) | 4 (50) | 1 (13) | 0 | 0 |
| Pyrexia | 3 (38) | 0 | 3 (38) | 0 | 0 | 0 |
| Chills | 2 (25) | 0 | 2 (25) | 0 | 0 | 0 |
| Fatigue | 2 (25) | 2 (25) | 0 | 0 | 0 | 0 |
| Ear pain | 1 (13) | 1 (13) | 0 | 0 | 0 | 0 |
| Encephalopathy | 1 (13) | 0 | 1 (13) | 0 | 0 | 0 |
| Erythema | 1 (13) | 0 | 1 (13) | 0 | 0 | 0 |
| Face edema | 1 (13) | 1 (13) | 0 | 0 | 0 | 0 |
| Heart rate increased | 1 (13) | 0 | 1 (13) | 0 | 0 | 0 |
| Hypophosphatemia | 1 (13) | 0 | 1 (13) | 0 | 0 | 0 |
| Hypotension | 1 (13) | 0 | 0 | 1 (13) | 0 | 0 |
| Nausea | 1 (13) | 0 | 1 (13) | 0 | 0 | 0 |
| Sinus tachycardia | 1 (13) | 1 (13) | 0 | 0 | 0 | 0 |
| Somnolence | 1 (13) | 0 | 1 (13) | 0 | 0 | 0 |
| Tremor | 1 (13) | 1 (13) | 0 | 0 | 0 | 0 |
| Visual impairment | 1 (13) | 0 | 1 (13) | 0 | 0 | 0 |
| Data cutoff date: February 14, 2022.  Percentages were calculated using the total number of patients treated with any dose of KITE-439 cells. TRAEs were coded using MedDRA version 24.1 and graded per CTCAE 5.0. Multiple incidences of the same AE in 1 patient were counted once at the worst grade for the patient.  CTCAE, Common Terminology Criteria for Adverse Events; MedDRA, Medical Dictionary for Regulatory Activities; TRAE, treatment-related adverse event. | | | | | | |

| Table S2. AESI by worst grade (N=8) | | | | | | |
| --- | --- | --- | --- | --- | --- | --- |
| **n (%)** | **Any** | **Worst**  **Grade 1** | **Worst**  **Grade 2** | **Worst**  **Grade 3** | **Worst**  **Grade 4** | **Worst**  **Grade 5** |
| **CRS*** | **3 (38)** | **2 (25)** | **1 (13)** | **0** | **0** | **0** |
| Pyrexia | 3 (38) | 0 | 3 (38) | 0 | 0 | 0 |
| Chills | 2 (25) | 0 | 2 (25) | 0 | 0 | 0 |
| Heart rate increased | 1 (13) | 0 | 1 (13) | 0 | 0 | 0 |
| Hypotension | 1 (13) | 0 | 0 | 1 (13) | 0 | 0 |
| Sinus tachycardia | 1 (13) | 1 (13) | 0 | 0 | 0 | 0 |
| **NEs** | **4 (50)** | **1 (13)** | **3 (38)** | **0** | **0** | **0** |
| Confusional state | 2 (25) | 1 (13) | 1 (13) | 0 | 0 | 0 |
| Depressed level of consciousness | 1 (13) | 0 | 1 (13) | 0 | 0 | 0 |
| Encephalopathy | 1 (13) | 0 | 1 (13) | 0 | 0 | 0 |
| Restlessness | 1 (13) | 1 (13) | 0 | 0 | 0 | 0 |
| Somnolence | 1 (13) | 0 | 1 (13) | 0 | 0 | 0 |
| Tremor | 1 (13) | 1 (13) | 0 | 0 | 0 | 0 |
| Data cutoff date: February 14, 2022.  *Overall CRS was graded per the revised grading system proposed by Lee et al^1^ and individual CRS symptoms were graded per CTCAE 5.0.  AEs are those with onset on or after initiation of the KITE-439 cell infusion. AEs were coded using MedDRA version 24.1 and graded per CTCAE version 5.0. Patients with multiple AEs of the same event of interest or preferred term were counted once at the worst grade for the patient. Percentages were calculated using the total number of patients treated with any dose of KITE-439 cells.  AE, adverse event; CRS, cytokine release syndrome; CTCAE Common Terminology Criteria for Adverse Events; NE, neurologic event. | | | | | | |

| Table S3. CD4+ and CD8+ T cell frequency in apheresis versus KITE-439 products | | | | |
| --- | --- | --- | --- | --- |
|  | **Apheresis** | | **KITE-439** | |
|  | **CD4+**  **(n=8)** | **CD8+ (n=8)** | **CD4+ (n=8)** | **CD8+ (n=8)** |
| Minimum | 50.48 | 15.91 | 24.99 | 47.45 |
| 25% Percentile | 57.20 | 24.60 | 28.50 | 53.39 |
| Median | 63.85 | 27.56 | 32.11 | 63.85 |
| 75% Percentile | 69.74 | 35.15 | 43.29 | 67.61 |
| Maximum | 77.85 | 44.71 | 51.40 | 72.08 |


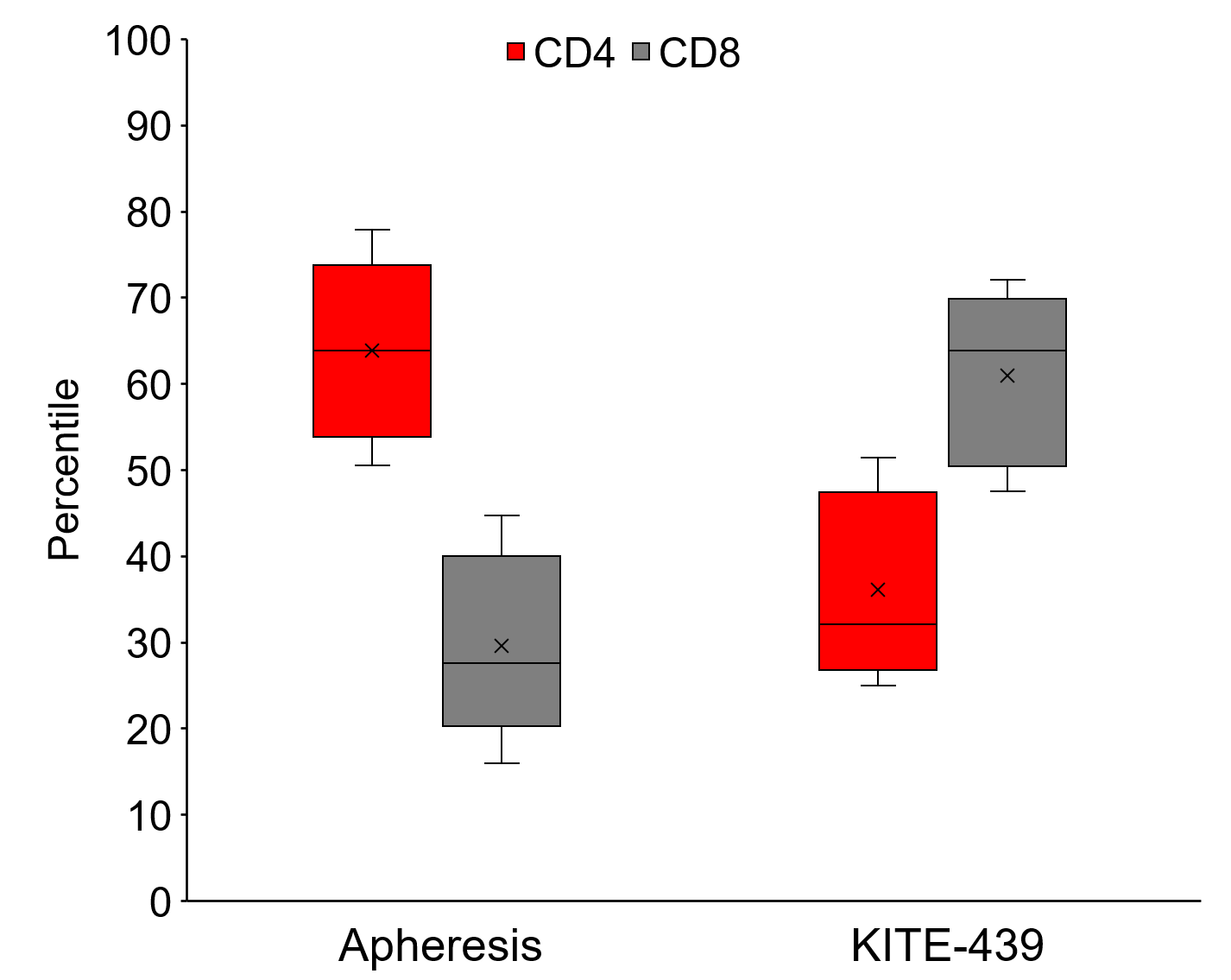


| Table S4. Analysis of *HLA-A*02:01* allele, *B2M*, and HPV oncogene status | | | | | | | | | |
| --- | --- | --- | --- | --- | --- | --- | --- | --- | --- |
|  | | **Cohort/Patient** | | | | | | | |
|  |  | **1** | **2** | **3** | **4** | **5-1** | **5-2** | **5-3** | **6** |
| **HLA somatic mutations** | ***B2M*** | No variant detected | No variant detected | No variant detected | No variant detected | No variant detected | No variant detected | No variant detected | No variant detected |
| **HLA allele-specific deletions** | ***HLA-A*2:01:01*** | No  deletions | No deletions | Deleted | No deletions | No deletions | No deletions | No  deletions | No deletions |
| **HPV oncogene status** | **Virus Identified** | HPV16 | HPV16 | HPV16 | HPV16 | HPV16 | HPV16 | HPV16 | HPV16 |

# SUPPLEMENTAL FIGURES

## Figure S1. Study design


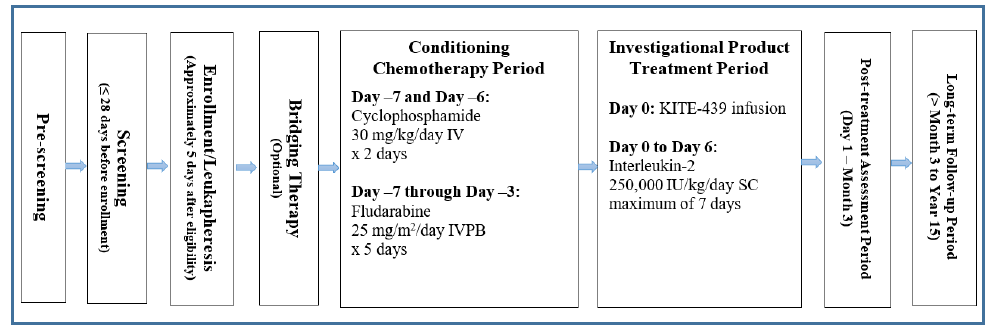

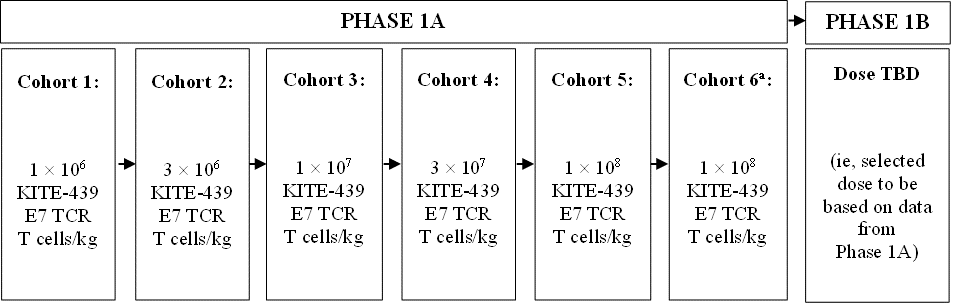


## Figure S2. Patient disposition

**
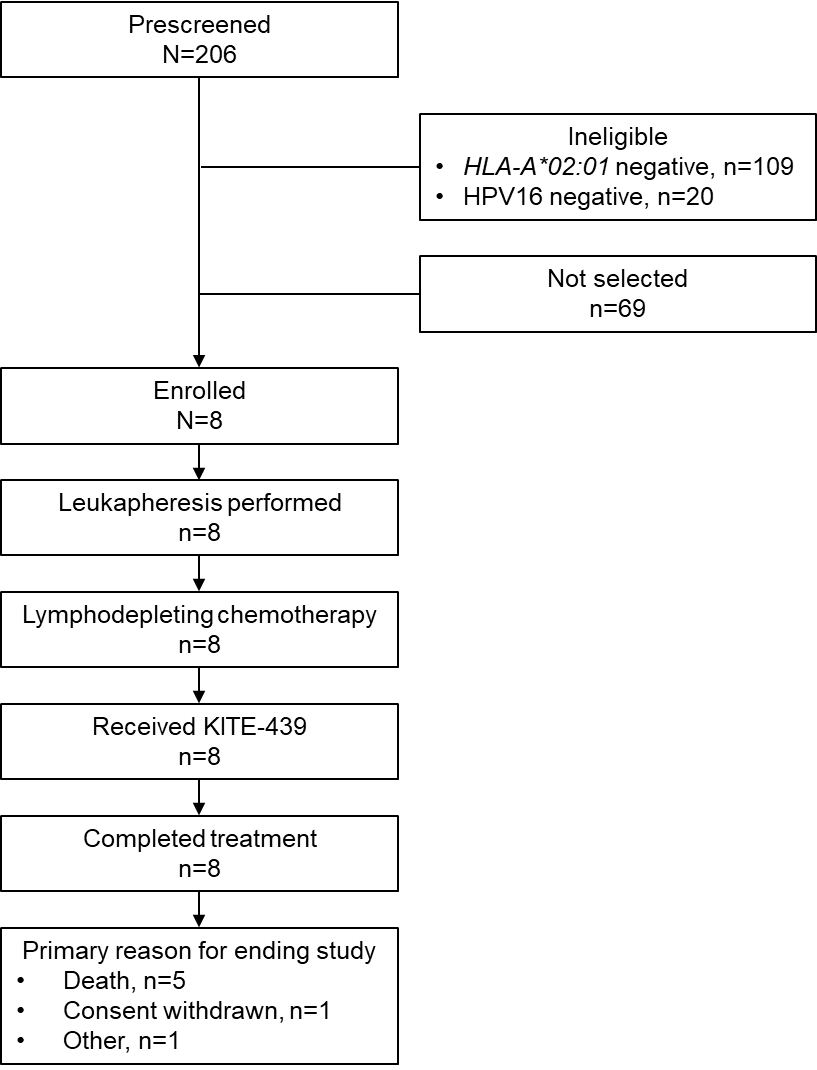
**

## Figure S3. Phenotypic evaluation of apheresis material and KITE-439 infusion products.

## Figure S4. T-cell phenotype in peripheral blood pre- and post-KITE-439 infusion

## Figure S5. Frequency of KITE-439 cells in peripheral blood and pleural fluid in the Cohort 4 patient (lung metastasis)

| **Peripheral Blood**  **(15.7% KITE-439 cells)** | **Pleural Fluid (13.9% KITE-439 cells)** |
| --- | --- |


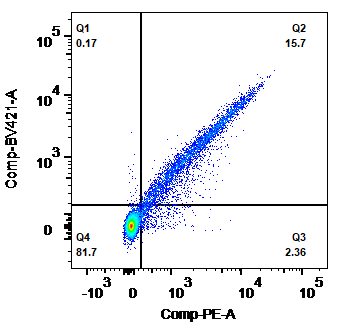

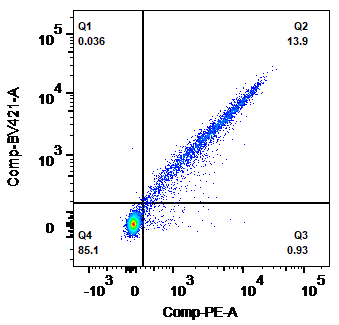


**KITE-493**

**cells**

**KITE-493**

**cells**

# SUPPLEMENTAL REFERENCES

1. Lee DW, Gardner R, Porter DL, et al. Current concepts in the diagnosis and management of cytokine release syndrome. *Blood*. 2014;124(2):188-95.

2. Nagarsheth NB, Norberg SM, Sinkoe AL, et al. TCR-engineered T cells targeting E7 for patients with metastatic HPV-associated epithelial cancers. *Nature medicine*. 2021;27(3):419-425.

3. Locke FL, Neelapu SS, Bartlett NL, et al. Phase 1 results of ZUMA-1: a multicenter study of KTE-C19 anti-CD19 CAR T cell therapy in refractory aggressive lymphoma. *Mol Ther*. 2017;25(1):285-295.

4. Topp MS, van Meerten T, Houot R, et al. Earlier corticosteroid use for adverse event management in patients receiving axicabtagene ciloleucel for large B-cell lymphoma. *Br J Haematol*. 2021;195(3):388-398.
